# West Nile virus transmission and human infection risk in Veneto (Italy): a modelling analysis

**DOI:** 10.1038/s41598-018-32401-6

**Published:** 2018-09-18

**Authors:** Giovanni Marini, Roberto Rosà, Andrea Pugliese, Annapaola Rizzoli, Caterina Rizzo, Francesca Russo, Fabrizio Montarsi, Gioia Capelli

**Affiliations:** 1 Department of Biodiversity and Molecular Ecology, Research and Innovation Centre, Fondazione Edmund Mach, San Michele all’Adige (Trento), Italy; 2 Epilab-JRU, FEM-FBK Joint Research Unit, Province of Trento, Italy; 3 Department of Mathematics, University of Trento, Trento, Italy; 4 Istituto Superiore di Sanità, Roma, Italy; 5 Regione Veneto, Venezia, Italy; 6 Laboratory of Parasitology, Istituto Zooprofilattico Sperimentale delle Venezie, Padova, Italy

## Abstract

An intensified and continuous West Nile virus (WNV) spread across northern Italy has been observed since 2008, which caused more than one hundred reported human infections until 2016. Veneto is one of the Italian regions where WNV is considered endemic, and the greatest intensity of circulation was observed during 2013 and 2016. By using entomological data collected across the region in those years, we calibrated a temperature-driven mathematical model through a Bayesian approach that simulates the WNV infection in an avian population with seasonal demography. We considered two alternative routes of life cycle re-activation of the virus at the beginning of each vector breeding season: in the first one the virus is maintained by infected birds, in the other by diapausing mosquitoes previously infected. Afterwards, we computed seasonal risk curves for human infection and quantified how they translate into reported symptomatic cases. According to our results, WNV is more likely to be re-activated each year via previously infected mosquitoes. The highest probability of human infection is expected to occur in August, consistently with observations. Our epidemiological estimates can be of particular interest for public health authorities, to support decisions in term of designing efficient surveillance plans and preventive measures.

## Introduction

West Nile virus (WNV) is a neurotropic mosquito-borne virus belonging to the Flavivirus genus and Japanese encephalitis virus serogroup^1^. WNV is maintained by an enzootic cycle involving birds and ornithophilic mosquitoes such as species of the *Culex* genus of which the *Culex pipiens* complex is thought to be one of the most important vectors in Europe^2^. Some passerine birds are among the most competent amplifier hosts since they develop sufficient serum viremia to infect efficiently mosquitoes feeding upon them^3,4^. Humans, horses and other mammals are dead-end hosts that may be incidentally involved in the enzootic cycle. While most human infections are asymptomatic, about 25% of the infections develop symptoms such as fever and headache^5^ and less than 1% more severe neurological diseases^6^.

WNV is a significant burden for public health in Europe, as it caused hundreds of cases during the last decade^7^. In Italy, WNV has caused severe illnesses in humans every season in many different regions. Despite its substantial impact, some of WNV epidemiological and ecological features have not completely elucidated yet. For instance, it is not known what proportion of persons develop infection following an infected mosquito bite^8^. While WNV is considered endemic in many parts of Europe, its persistence mechanism is still under discussion; it might survive from one season to the next in resident avian hosts^9,10^, mosquitoes^11^ or it might be reintroduced each year with migratory birds^12^.

WNV was detected in Italy for the first time in 1998, during an outbreak among horses in Tuscany^13^, and then re-emerged ten years later in the northeastern part of the country. Though first detections belonged to lineage 1 (the Western Mediterranean clade), WNV lineage 2 (WNV-2) is nowadays the most frequently identified lineage in Italy^14^. The first human infection due to WNV-2 was reported in Central Italy in 2011^15^ and subsequently WNV-2 became the only strain isolated in humans and mosquitoes in Italy^16^. Recent phylogenetic investigations show that WNV-2 probably entered Italy in 2007 from the east Adriatic coast and reached the northeastern part of the country in 2011^17^, with a peak of human cases in 2013^14^. WNV cases have been recorded each year across the country, with peaks occurring between August and September^18,19^.

In this study, we examine WNV transmission in Veneto region (northeastern Italy), where it is now considered endemic. In fact, WNV has been detected in *Cx. pipiens* mosquitoes^20^ and a number of cases of human or horse/bird infections are reported every year within the region^16^. In particular, we focus on determining the most likely route of life cycle re-activation, i.e. whether WNV transmission starts each year with already infectious birds or mosquitoes; in this latter case mosquitoes were possibly infected the previous year and overwintered in a diapausing state. To this aim, we develop a mechanistic temperature-driven model that replicates the avian-vector transmission cycle. While following the structure of previously published studies (e.g^21,22^.), our modelling effort relies on some important novelties. Indeed, it is calibrated on the vector prevalence recorded in the study area over multiple years and the mosquito dynamics is not modelled explicitly but it is approximated using field observation. Using our modelling results, we estimate the risk of human transmission during the mosquito breeding season (May-October). Finally, we fit the observed human cases to quantify how the human transmission risk predicted by the model translates into reported symptomatic cases.

## Methods

### Study area and entomological data

**Figure 1:**
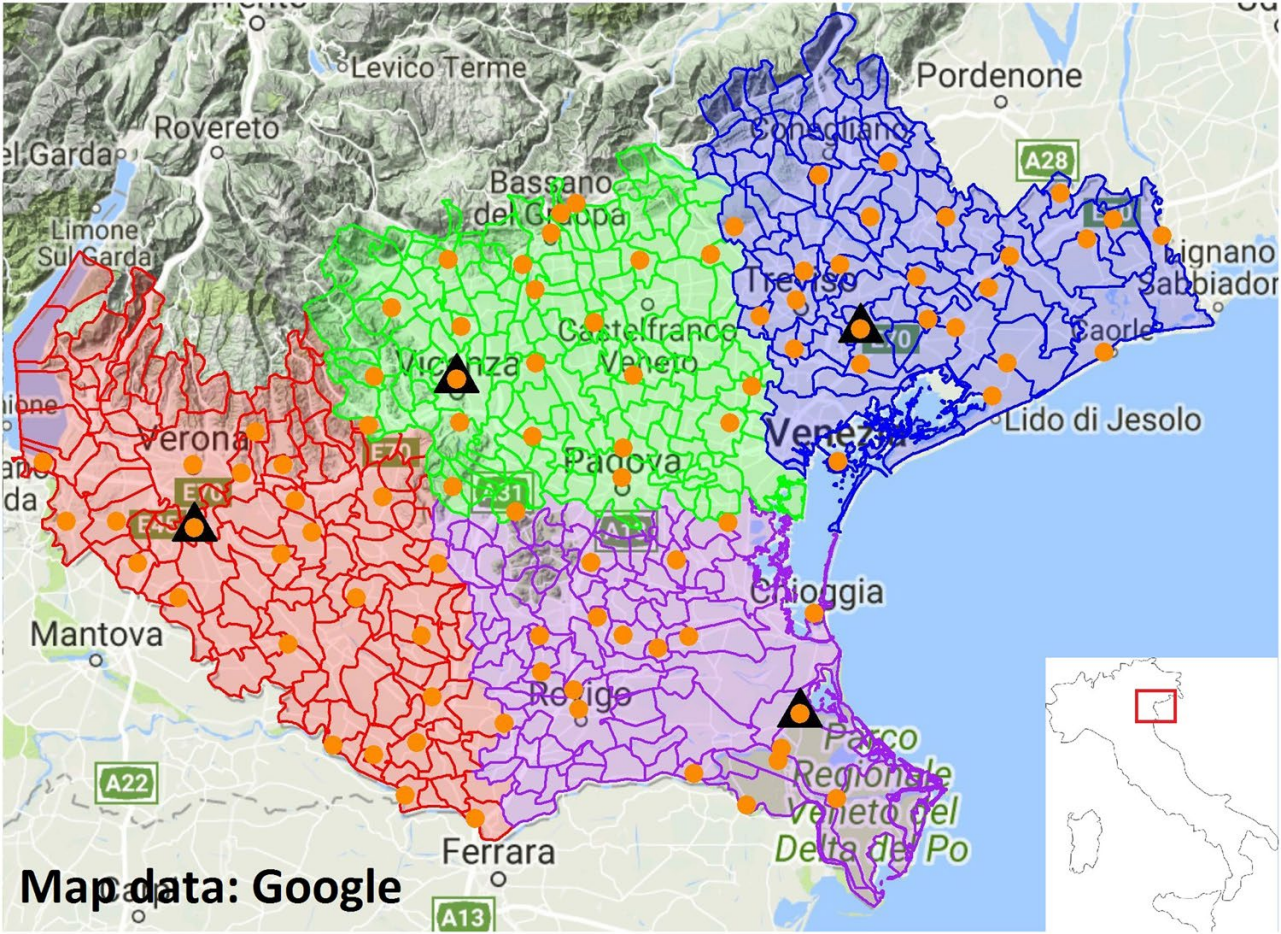
Study area. Orange points represent the traps location. Municipality boundaries are displayed in red, green, purple and blue according to the cluster they belong to. Weather stations locations are indicated with black triangles. Map data: Google.

The study area is located in the plain part of Veneto region (northeastern Italy, average altitude 77 m) and includes the provinces of Padova, Rovigo, Treviso, Venezia, Verona and Vicenza. A total of 109 trap locations (orange points, Fig. 1) were chosen among 92 municipalities. Mosquitoes were collected from May to October 2013–2016, using CDC traps baited with CO_2_. Once every two weeks, each trap was set in the morning and checked after 24 hours. After identification, individuals belonging to the *Cx. pipiens* species were pooled (average pool size of 50 mosquitoes) to detect the presence of WNV.

We divided the study area in 4 separated clusters (colored regions in Fig. 1) according to their locations by the k-means clustering method^23^. The four clusters’ extension range from 2,719 km^2^ to 3,527 km^2^, with population ranging from 574,000 to 1,696,507 inhabitants.

Temperature data for each cluster, collected with ground stations, was obtained from ARPA Veneto^24^. As all clusters exhibit very similar landscape characteristics, they also show very similar temperature patterns during the year (see Supplementary Material). The average temperature for the study period (May-October) is 20.3 °C, with maximum and minimum daily records of 30.6 °C in 2015 and 7.2 °C in 2014 respectively.

### WNV model

For each cluster, we approximated the average *Cx. pipiens* weekly captures with a smooth spline. Thus, after rescaling them by the trapping capture rate, α, we obtained for any day *t* between May 1 and October 31 an average adult mosquito density *M*(*t*) for an average trapped area *A* for every considered cluster, with *A* = π∙*r*^2^, where *r* is the average *Cx. pipiens* flight range.

**Figure 2:**
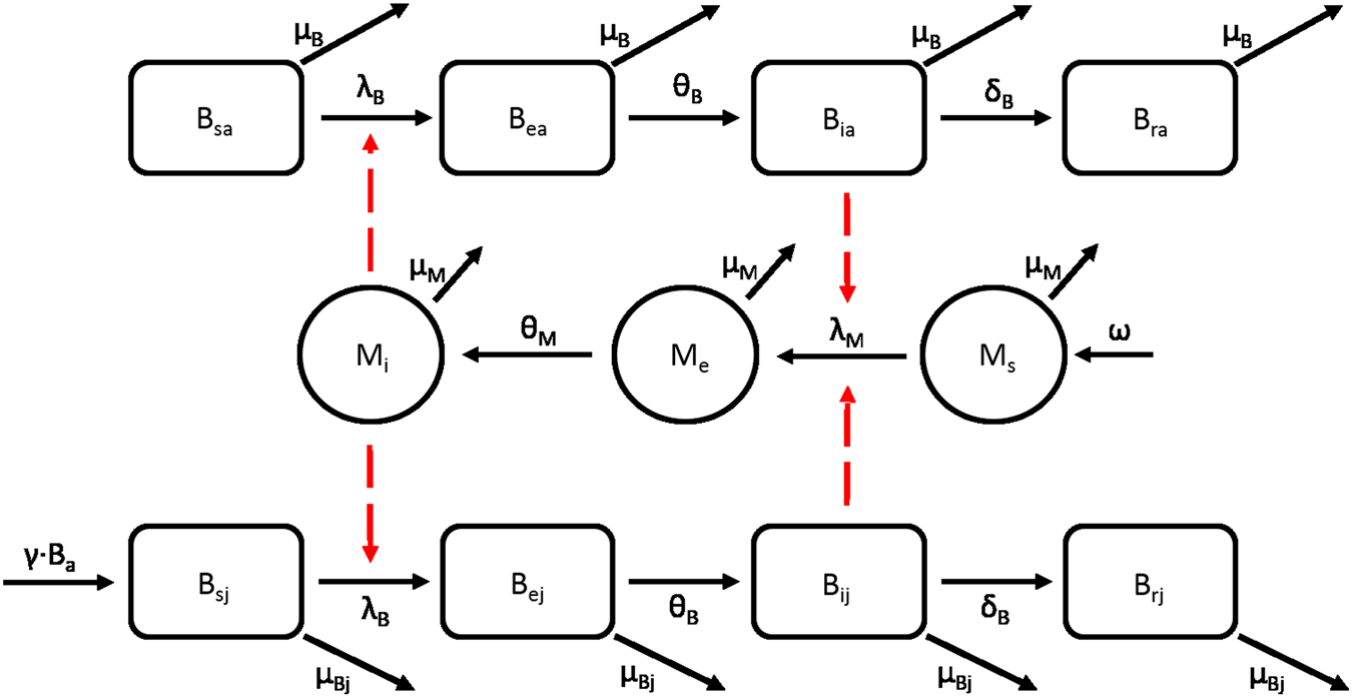
Model scheme. Model flow chart for WNV transmission in birds (squares) and mosquitoes (circles) in an average trapped area. Compartments: B_sa_, B_ea,_ B_ia,_ B_ra_ (B_sj_, B_ej,_ B_ij,_ B_rj_): adult (juvenile) susceptible, exposed, infectious and immune birds; M_s_, M_e_, M_i_: susceptible, exposed and infectious mosquitoes. Parameters: λ_B_ and λ_M_ are the force of infection for birds and mosquitoes respectively and are computed as λ_B_ = *b*∙p_MB_∙M_i_/B_T_ and λ_M_ = *b*∙p_BM_∙(B_ia_ + B_ij_)/B_T_, with B_T_ being the total avian population and B_a_ the number of adult birds. See Table 1 for parameter values and references.

We modelled the transmission of WNV within the average trapped area *A* among an initially fully susceptible avian community during a typical season (i.e. from May 1 to October 31) according to the compartmental scheme reported in Fig. 2 (model equations can be found in the Supplementary Material). The biological interpretation of model parameters and their values is summarized in Table 1 (see also the Supplementary Material for more details). For the sake of simplicity, we consider all WNV-competent birds as belonging to a single species, whose parameters correspond to those of the House sparrow (*Passer domesticus*), a common species in the area^25^ which is competent for WNV transmission^4^ and is considered to be one of the most important avian reservoirs for the virus^26^. At the beginning of the season (May 1) the bird community is assumed to consist of adult individuals only, which can breed and reproduce until mid-July, giving birth to juvenile individuals. As maturity age is one year^27^, they are considered as juveniles throughout the entire season. Susceptible adult (juvenile) birds B_sa_(B_sj_) contract the virus from bites of infectious mosquitoes. After an intrinsic incubation period, they become infectious and subsequently recover and become immune to reinfections. We did not consider possible deaths due to the infection as laboratory experiments show a low or even no mortality for this species when infected with European WNV strains^4^ and very limited wild bird mortality due to WNV is usually observed in Europe^2^. Susceptible mosquitoes (M_s_) can become exposed to infection (M_e_) after biting infectious birds with a temperature-dependent probability (see Table 1); in such a case, they will become infectious to the avian population (M_i_) after a temperature-dependent extrinsic incubation period and for the rest of their life. We neglected vertical transmission as it occurs at a very low rate^28,29^ and is probably relevant only for infection persistence between seasons^30,31^.

**Table 1:** Model parameters. T denotes the daily average temperature (°C).

Parameter	Explanation	Value	Source
μ_M_	Mosquito death rate (day^−1^)	$\frac{4.61}{151.6-4.57\cdot {\rm{T}}}$	^56, 57^
α	Mosquito capture rate (day^−1^)	0.054	^58^
*r*	*Cx. pipiens* daily flight range (meters)	500	^59, 60^
p_MB_	Probability of WNV transmission from mosquito to bird per infectious bite	0.94	^4^
p_BM_	Probability of WNV transmission from bird to mosquito per infectious bite	$\frac{{{\rm{e}}}^{(-10.917+0.365\cdot {\rm{T}})}}{1+{{\rm{e}}}^{(-10.917+0.365\cdot {\rm{T}})}}$	^61^
θ_M_	Extrinsic incubation period (day^−1^)	$-0.132+0.0092\cdot {\rm{T}}$	^62^
θ_B_	Intrinsic incubation period (day^−1^)	0.5	^4^
δ_B_	Avian recovery rate (day^−1^)	0.57	^4^
γ(t)	Avian fertility rate at day *t* (day^−1^)	0.05 (*t* ≤ July 15) 0 (*t* > July 15)	^63^
μ_B_	Death rate for mature birds (day^−1^)	0.0015	^63^
μ_Bj_	Death rate for juvenile birds (day^−1^)	0.0083	^63^

We assume that mosquitoes die at temperature-dependent rate μ_M_, but we do not model completely mosquito population dynamics, as it will depend in a complex way on several environmental variables. Rather we consider the total number of adult mosquitoes, *M*(*t*), obtained from the spline, as a known function; to this aim, if ω(*t*) = *M*(*t*) − (M_s_(*t*) + M_e_(*t*) + M_i_(*t*)) > 0, then at day *t* new ω(*t*) susceptible mosquitoes enter the system.

As stated above, we model independently every breeding season and therefore we need the virus to be already present at its beginning. For the sake of simplicity, we considered two alternative routes of re-activation of the virus: B) the virus is alive in infected birds (B_i_(0) > 0, M_i_(0) = 0); M) some initial mosquitoes are already infectious (B_i_(0) = 0, M_i_(0) > 0). To assess which hypothesis explained better the observed data, we compared the goodness of fit of the two modelling scenarios using the Deviance Information Criterion (DIC)^32^.

The initial number of adult birds B_0_(y), the initial prevalence *p*(y) (either in birds or in mosquitoes, according to the considered scenario), which are year-dependent, and the biting rate *b*, which is assumed to be equal among years, are the set of unknown parameters Ψ (i.e. Ψ = {B_0_(2013), B_0_(2016), *p*(2013), *p*(2016), *b*}. The posterior distributions of Ψ were explored by Markov chain Monte Carlo (MCMC) sampling applied to the binomial likelihood of observing the recorded number of positive pool, given the model-predicted mosquito prevalence. Assuming that for each week the number of observed positive follows a binomial distribution B(N, P) where N is the number of tested pool and P the probability that a pool is positive obtained from the model, the likelihood of the observed data over the two simulated years has been defined as

$$L=\prod _{y\in \{2013,\,2016\}}\prod _{s=1}^{{N}_{s}(y)}(\begin{array}{c}N(s,\,y)\\ K(s,\,y)\end{array})P{(s,y,{\rm{\Psi }})}^{K(s,y)}{(1-P(s,y,{\rm{\Psi }}))}^{N(s,y)-K(s,y)}$$

Where *y* and *s* run over the considered years and sampling dates respectively, N_s_(*y*) is the number of samplings for year *y*, K(*s*, *y*) is the number of recorded positive pools for sampling *s* and year *y* and

$$P(w,y,{\rm{\Psi }})=1-{(1-\frac{{M}_{i}(s,{\rm{\Psi }})}{M(s,{\rm{\Psi }})})}^{u(s)},$$

where M_i_(s, Ψ) and M(s, Ψ) are respectively the number of infected and total mosquitoes predicted by the model for the sampling date *s* with parameters Ψ and u(*s*) is the average pool size. The posterior distribution of Ψ was obtained by using random-walk Metropolis-Hastings sampling approach and normal jump distributions.

Finally, we performed a sensitivity analysis by exploring how different values for the model constant parameters, namely p_MB_, δ_B_, θ_B_, μ_B_, μ_Bj_ and γ, might affect the model results. We evaluated the effect of these perturbations both on the predicted avian and mosquito infection prevalence and on the estimated posterior distribution of the free model parameters (see Supplementary Material).

### Human infections

On average, about *f* = 6.9% of *Cx. pipiens* blood meals are taken from human hosts in the study area^25^. We estimated the probability λ_H_(*w*) for a human living in a cluster to be bitten by a WNV infected mosquito in any week *w* during the study season as follows: we divided the estimated number of daily infectious vector bites on humans, obtained as *f*∙*b*∙M_i_(w) where *b* is the previously estimated biting rate, by the number of humans living in the study area *A*. We also allowed the vector to shift the feeding preference during each year^33^. Specifically, we let *f* vary between early and late summer as reported in^25^, where the authors found that the percentage of *Cx. pipiens* blood meals on human increased from 3.6% to 8.3% after June 30.

Finally, we investigated which scenario (B or M with or without a shifting mosquito feeding preference) better explains the symptomatic human infections observed in a cluster, by predicting the number of reported WNV human infections N_w_ from a Poisson(H∙ρ∙ λ_H_(*w*)), where H is the number of human beings living in a cluster and ρ is a free rescaling parameter, estimated with a MCMC approach applied to the Poisson likelihood of observing the recorded infections, given the model predictions, by modelling together the considered years. We can interpret ρ as a product of the probability of virus transmission to humans per mosquito infectious bite times the probability of symptoms development times the reporting rate.

## Results

### Cluster analysis

The number of WNV positive pools found in the study area greatly varied between years, from a minimum of 3 in 2015 to a maximum of 57 in 2013. We decided to focus our analysis on 2013 and 2016 (27 WNV positive pools), the two years with most positive pools and highest human incidence^19^. Mosquito trapping season started on May 7 and 20 in 2013 and 2016 respectively. Moreover, we carried out model simulations only for the cluster that includes the province of Verona and partially of Rovigo (in red in Fig. 1, area 3,527 km^2^, 1,016,138 inhabitants) as the great majority of the positive pools were collected in that area (respectively 50 and 22 for the two years). The interpolated mosquito abundance for this cluster in the two study years is shown in the Supplementary Material.

### Model fit

**Figure 3:**
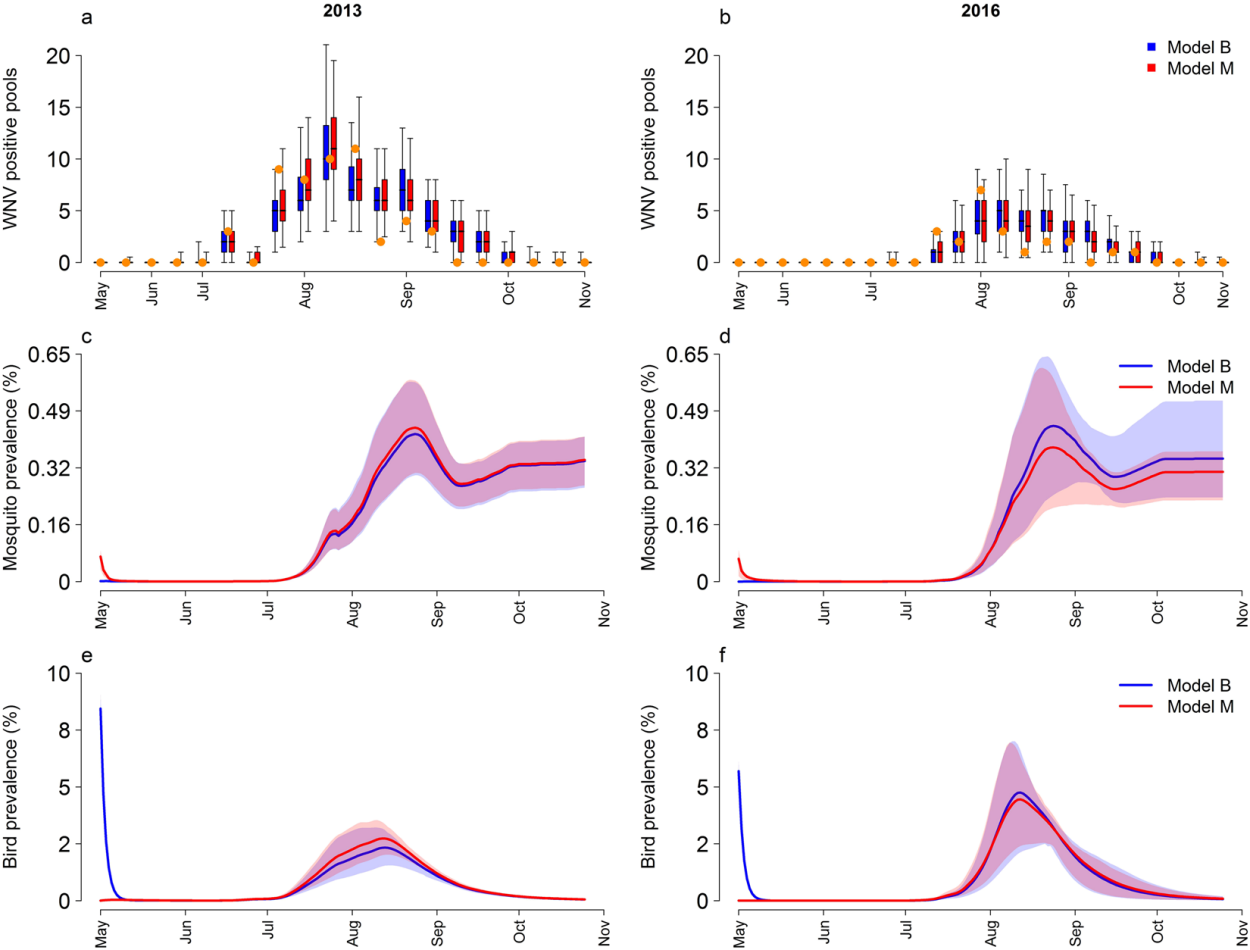
Model predictions. Predicted number of WNV positive pools (panels a, b) mosquito prevalence (panels c, d) and avian prevalence (panels e, f) for 2013 (first column) and 2016 (second column) according to model assumption: blue: model B; red: model M. Panels a-b: orange points: observed weekly number of WNV positive pools; boxplots (median, quartiles and 95% quantiles) show the predicted distributions of positive pools per week. Panels c-f: solid lines: average values; shaded regions: 95% credible interval.

As shown in Fig. 3 (first row) the model fit is qualitatively very similar between the models [B (M): some initial birds (mosquitoes) are already infectious at season start]. The DIC values are 109.23 and 104.03 for scenario B and M respectively, thus the hypothesis that the virus is introduced at the beginning of the season via infected mosquitoes seems significantly more likely. In both cases, we can note that the mosquito prevalence (second row, Fig. 3) starts to increase in June and reaches in maximum in August. Then it declines and remains stable from October onwards. Unsurprisingly, the mosquito prevalence in May is a little higher in scenario M than in B, although it is very low compared to the highest values occurring later in summer. Conversely, the simulated avian prevalence in May is very high for model B (Table 2), and even higher than in summer (Fig. 3, blue lines in panels e and f). These patterns are confirmed by the sensitivity study on model constant parameters (see Supplementary Material).

**Table 2:** Estimated model parameters distributions (average and 95% credible intervals).

Parameter	Model B	Model M
*b*	0.23 (95%CI 0.19–0.31)	0.2 (95%CI 0.17–0.22)
B_0_ (2013)	110 (95%CI 72–207)	85 (95%CI 63–109)
B_0_ (2016)	28 (95%CI 20–55)	25 (95%CI 20–33)
*p* (2013)	0.15 (95%CI 0.14–0.16)	7.7∙10^–4^ (95%CI 3.8∙10^–4^–9.6∙10^–4^)
*p* (2016)	0.10 (95%CI 0.09–0.11)	6.8∙10^–4^ (95%CI 2.3∙10^–4^–9.4∙10^–4^)

At the end of the season, the fraction of recovered birds (B_ra_ + B_rj_)/B_T_ is on average between 0.66 and 0.92 (see Supplementary Material). In particular, it is lower in 2013 than in 2016 regardless of the initial hypothesis. In the case of model M, this fraction starts to increase exponentially in July, as avian prevalence increases, reaching a steady level in September. In the case of model B, recovered birds are about 10% of the total avian community in June already, in line with estimated initial prevalences (Table 2).

The estimated posterior distributions for the biting rate are quite close for the two modelling assumptions (Table 2). Conversely, the estimated number of initial birds is lower for model M and in both models the avian population for 2016 is expected to be smaller than in 2013. We can note that the estimated initial prevalence is much higher for birds than for mosquitoes. Finally, perturbations of the model constant parameters do not produce substantial variations of posterior distributions (see the sensitivity analysis within the Supplementary Material).

### Human infections

In the considered cluster, 16 and 8 human WNV symptomatic infections were recorded in 2013 and 2016 respectively, with dates of symptoms onset ranging from mid-July to the end of September.

**Figure 4:**
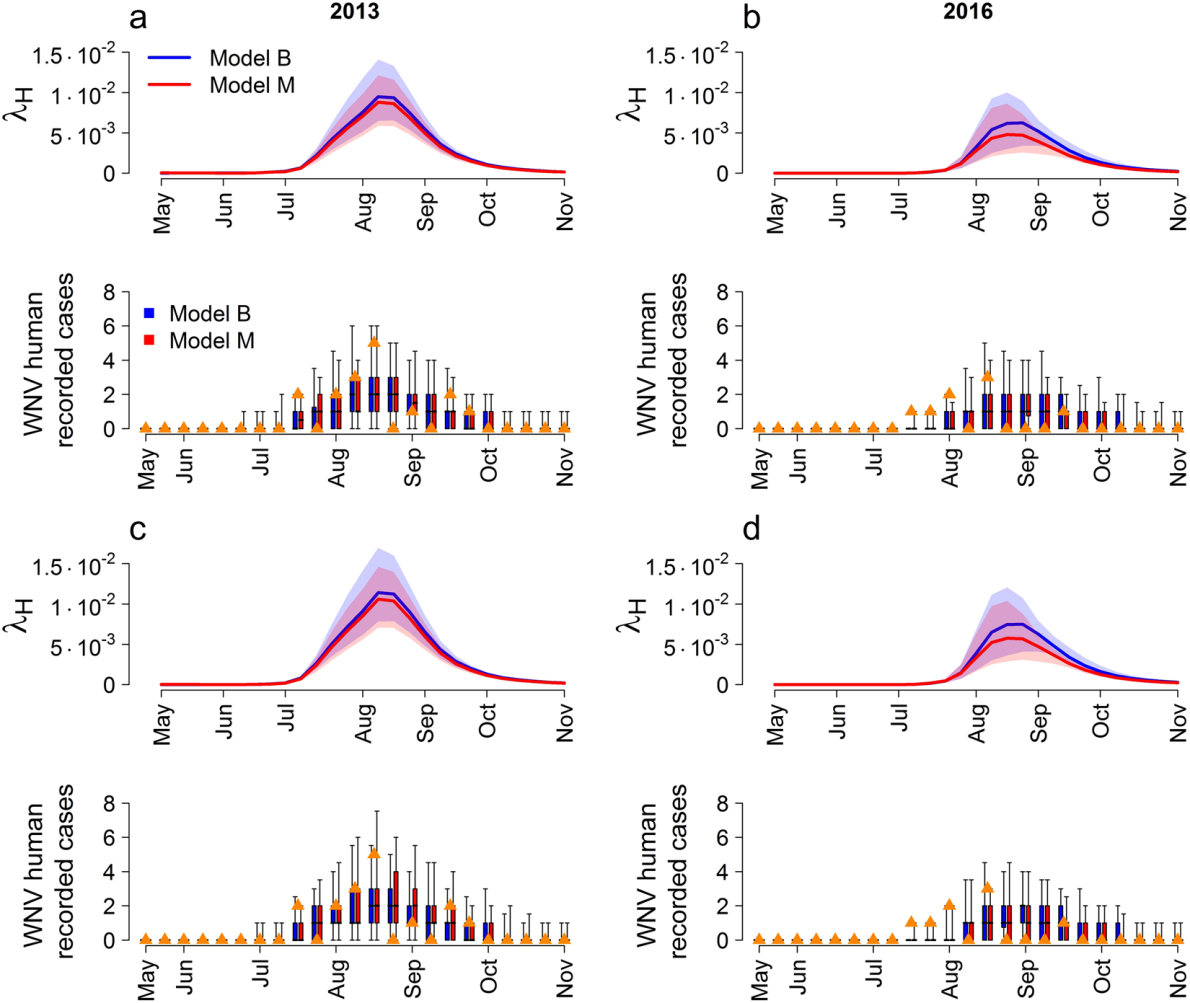
Human transmission risk and infections. Estimated weekly probability per person of being bitten by a WNV infected mosquito (λ_H_, first row of each panel) and notified human symptomatic cases (second row of each panel) in 2013 (first column) and 2016 (second column). Blue: the virus is introduced with birds (model B). Red: the virus is introduced with mosquitoes (model M). Panels a, b: *f* = 5.9%. Panels c, d: *f* = 3.6% until June 30, afterwards *f* = 8.3%. Solid lines: average value. Shaded regions: 95% credible interval. Orange triangles: observed WNV symptomatic cases per week of symptoms onset. Boxplots (median, quartiles and 95% quantiles): predicted symptomatic recorded cases.

As shown in Fig. 4 (first row of each panel), the predicted risk λ_H_ for a human living in the considered cluster of being bitten by an infectious mosquito in one week varies substantially between the two years (accordingly to the higher proportion of positive pools recorded in 2013 than in 2016), and they are higher when assuming time-dependent feeding preferences. In fact, the highest average probabilities, about 0.011 and 0.007, occur at the beginning of August in 2013 and 2016 respectively by allowing *f* to take different values before and after June 30 (panels c, d). In general, we can note that from late July to the end of August humans are at greater risk of WNV transmission, consistently with observations. Remarkably, the model predicts a halved risk for 2016 with respect to 2013, following the same pattern observed for the number of cases.

Figure 4 (second row of each panel) shows the fit of the observed number of WNV symptomatic cases for the two years. It is clear that different model assumptions produce fits that are qualitatively very similar, and in fact the associated DIC values are very close (Table 3). Anyhow, we can note that the lowest DIC value is achieved assuming the virus is introduced with infected mosquitoes (model M) and *f* shifts from 3.6% to 8.3% after June 30. ρ estimates concur between different models, being on average between 1.3∙10^−3^ and 1.9∙10^−3^, meaning that between one and two out of 1,000 infectious mosquito bites on humans will result in a symptomatic reported case.

**Table 3:** Human cases fit. DIC values and estimated ρ distribution (average and 95% credible intervals) for the four modelling assumptions.

Model	DIC	ρ
Model B, fixed *f*	79.6	1.6∙10^−3^ (9.6∙10^−4^–2.4∙10^−3^)
Model M, fixed *f*	78.2	1.9∙10^−3^ (1.1∙10^−3^–3∙10^−3^)
Model B, time dependent *f*	79.9	1.3∙10^−3^ (7.9∙10^−4^–2∙10^−3^)
Model M, time dependent *f*	77.2	1.5∙10^−3^ (9.1∙10^−4^–2.3∙10^−3^)

## Discussion

In this study, we analysed the dynamics of WNV in Veneto region, Northern Italy. In particular, we investigated how the virus life cycle is re-activated every year in the area and we quantified the risk of human spillover.

Our modelling approach included the estimate of some unknown parameters, such as the avian population size and the mosquito biting rate. Assuming our model simulates an area *A* = π∙*r*^2^ = 0.79 km^2^ (*r* = 500 m), the computed initial House sparrow density spans between 30 and 140 individuals per km^2^. Although we do not have a precise estimate on the number of birds living in the area, we note that these estimates are consistent with other ranges obtained in different parts of the world^27^. Our results for the biting rate indicate *Cx. pipiens* has a gonotrophic cycle of about 5 days during the breeding season in the study region, very close to the measure published in^34^ (5.54 ± 1.73 days in laboratory conditions). To the best of our knowledge^34^, presents the only available data regarding the influence of temperature on *Culex pipiens* gonotrophic cycle. Since there are no data for different temperatures and many WNV modelling studies assume the biting rate to be temperature independent^21,22,31,35^, we decided to consider *b* as a free model parameter which is constant during the season.

According to our results, model fits obtained with different assumptions are qualitatively very similar. We computed DIC values for every model fit we performed, in order to assess quantitatively which assumptions better explained the observed values. The difference between the highest and lowest DIC values was 5.2 when fitting the number of observed mosquito positive pools. As the recommended DIC difference threshold is at least two^32^, assuming the virus is introduced at the beginning of the season by infected mosquitoes (model M) provides a significantly better fit. Conversely, when fitting the observed number of reported symptomatic human infections, different modelling assumptions produce very similar DIC values. Although there is no striking indication in favour of one particular model, we can note that the lowest DIC is given by model M coupled with shifting mosquito-feeding preferences.

Another observation that makes model B (WNV season starts with infected birds) unlikely is that the highest avian prevalence is predicted to occur in spring; this is contrary to observation, as usually WNV positive birds are found in summer^36^, and would require that most infection transmission would occur when the mosquito density is relatively very low. Indeed, it has been suggested that in North America WNV might persist in winter in American crow populations, which can transmit the virus with fecal-oral transfer^9,10^. Moreover, it has been demonstrated that WNV can persist in House sparrow tissues, and this may lead to oral transmission to predatory birds and other animals during times of interrupted mosquito activity^37^. Thus, winter roost sites might be potential points for vernal amplification of WNV once mosquito activity increases. This might be true also for Italy, but very few studies have been conducted to assess European avian species competence for this virus and the relative contribution of non vectorial transmission. Furthermore, our modelling study suggests that the prevalence in birds in spring should be very high to comply with the overall dynamics, and it seems difficult that it would go undetected. Some modelling studies suggest instead that the virus can be introduced in Europe every year through birds migrating from Africa^12,38^, but phylogenetic analyses of European and Italian WNV lineages strongly support the hypothesis that the virus overwinters in the area and its introduction occurred years ago^14,39–42^. Concerning the hypothesis that the virus overwinters through infected mosquitoes (model M), it is possible that vertically-infected diapausing mosquitoes, emerged at the end of the breeding season, initiate transmission in the following spring^11^ and the first detection of WNV in overwintering mosquitoes in Europe, occurred in 2017^43^, supports this hypothesis. We did not explicitly consider vertical transmission as it occurs sporadically^28,29^ and modelling the inter-seasons interval (i.e. November-April) was beyond our scope, also because of the lack of data regarding mosquito and bird survivorship in this period.

The average daily avian prevalence was estimated to be no higher than 5% during the summer months, a result similar to actual observations made in different areas. For instance, about 12% of House sparrow nestlings tested during August 2008 were found to be WNV infected in a rural area in the US^44^ while WNV prevalence in birds was observed to be as high as about 8% in August 2005 in Chicago^45^.

The final seroprevalence in birds was estimated between 66 and 92% on average. Reported values vary greatly among locations and years: in Spain in 2004, seroprevalence in blackbirds (*Turdus merula*) was 5.4%^46^, while for House sparrows it was estimated to be 1.96% in 2013 in the same study area^47^. On the other hand, in three close rural House sparrow colonies in the US about 27% of birds tested positive for WNV antibodies in 2008^44^, while seroprevalence was observed to be as high as about 30–40% in Los Angeles in October 2004 and 2009^48^ and 24.4% in Chicago between May and October 2005, with a peak value of more than 60% recorded at the end of September^45^. In Romania, 33.96% of wild resident birds tested positive between 2011 and 2012 after a big WNV outbreak (50 reported human cases) occurred in 2010^49,50^. The particularly high value obtained in our simulations might depend on our simplifying assumption that there is only one avian competent species. In reality there are several of them^3,4,51–53^, whose competence varies considerably. With more species, possibly less competent, WNV spread would be limited thanks to a dilution effect^54,55^. Lack of detailed ornithological data prevented us from designing a more realistic model. Moreover, we assumed that the avian population is fully susceptible at the start of the season; however, most immune birds will survive winter and therefore they will be present in the following year, thus reducing the number of potential hosts and of WNV infections during the season. This might be a possible explanation of the low WNV circulation observed in the cluster area in 2014 and 2015 (only seven and two positive pools respectively). This might also explain the lower estimate obtained for the initial number of susceptible birds in 2016.

The sensitivity analysis carried out on the constant parameters set enhances the robustness of our findings and the realism of model predictions. In fact, perturbations of model constant parameters produce very small variations in both model simulations and estimated free model parameters (see Supplementary Material).

After calibrating our WNV model, we estimated the human spillover risk. This probability is higher from mid-July to August, and it can be slightly higher than 0.01, meaning that about one in a hundred people will be bitten by an infectious mosquito in that particular week. The human risk was estimated to be lower for 2016, since fewer positive pools were collected with respect to 2013, in compliance with the smaller number of recorded cases. By fitting the reported WNV human infections, we computed the probability for a mosquito infectious bite to result in a reported human infection. To the best of our knowledge, there are no estimates for the transmission probability from mosquitoes to humans^8^, nor for the reporting rate for WNV infections, while the proportion of symptomatic infections is about 25%^5^. Thus, our result might be particularly useful to provide some estimate of the symptomatic human infections also in other similar regions.

In addition, we implemented a simpler fit of the observed human infections by using only the mosquito prevalence observed with the pools and the interpolated vector abundance, without explicitly modelling the avian-vector transmission cycle (see Supplementary Material). However, this simplified method yielded a much worse fit, both qualitatively and quantitatively. This negative result corroborates the relevance of our modelling approach, making our findings more useful and reliable.

Despite its limitations, our study provides new important insights on the ecology of WNV in Southern Europe, in particular regarding its endemism and seasonal dynamics. Estimated human infection risk during the season can be of particular interest for public health authorities, to support decision in designing efficient surveillance and prevention strategies. However, this study highlights the urgency to carry out more detailed eco-epidemiological studies on WNV host and vector interaction in Italy to obtain more precise estimate of the changing hazard and risk of transmission to humans.

## Electronic supplementary material

Supplementary Material

Dataset 1

## Data Availability

All data generated or analysed during this study are included in this published article (and its Supplementary Information files).
