# Supplementary material for: West Nile virus transmission and human infection risk in Veneto (Italy): a modelling analysis
Source: Sci Rep. 2018 Sep 18;8:14005. doi: 10.1038/s41598-018-32401-6 (PMC6143586; doi:10.1038/s41598-018-32401-6)
Supplement: Supplementary file 1 — Supplementary Material [file 41598_2018_32401_MOESM1_ESM.pdf]

Supplementary Information

West Nile virus transmission and human infection risk in Veneto (Italy): a modelling analysis

Giovanni Marini[1], Roberto Rosà[1,2], Andrea Pugliese[3], Annapaola Rizzoli[1,2], Caterina Rizzo[4], Francesca Russo[5], Fabrizio Montarsi[6], Gioia Capelli[6]

1) Department of Biodiversity and Molecular Ecology, Research and Innovation Centre, Fondazione Edmund Mach, San Michele all'Adige (Trento), Italy;
2) Epilab-JRU, FEM-FBK Joint Research Unit, Province of Trento, Italy;
3) Department of Mathematics, University of Trento, Italy;
4) Istituto Superiore di Sanità, Roma, Italy;
5) Regione Veneto, Venezia, Italy;
6) Laboratory of Parasitology, Istituto Zooprofilattico Sperimentale delle Venezie, Padova, Italy.

Average recorded temperatures

Temperature data for each cluster, collected with ground stations, was obtained from ARPA Veneto [1].

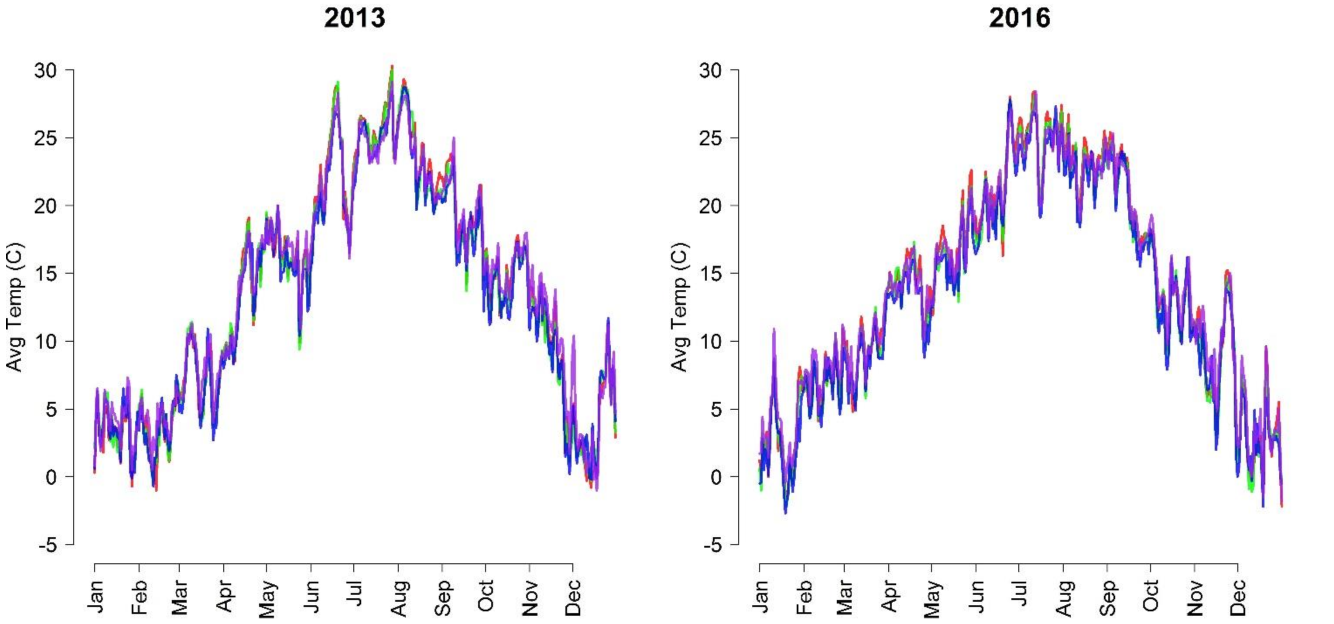

Supplementary Figure S1. Average temperature. Average daily temperature (°C) for the four clusters.

Mosquito abundance

We approximated the average *Cx. pipiens* weekly captures of the considered cluster (in red in Figure 1), rescaled for the capture rate α (see Tab. 1 in the main text), with a smooth spline, obtaining an average adult mosquito density $M(t)$ for each day t between May 1 and October 31. Thus, M(t) can be interpreted as the number of *Cx. pipiens* mosquitoes being present at day t in an area $A=\pi \cdot r^2$ of the considered cluster.

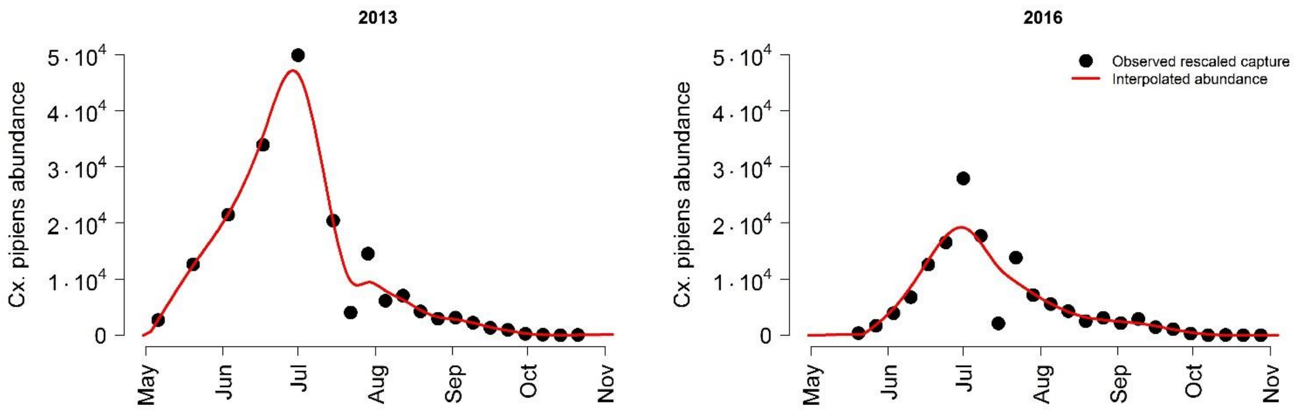

Supplementary Figure S2. Interpolated mosquito abundance (M(t)) for an average trapped area *A* of the considered cluster (in red in Figure 1 in the main text). Red lines: interpolated mosquito abundance. Black dots: observed average weekly captures rescaled by the capture rate α.

Additional methods

In this section, we provide additional details on the model and its temperature-dependent functions.

Model equations

The model flow depicted in Figure 2 in the main text is a visual description of the following set of equations:

$$
\begin{aligned}
&M_s'(t) = \omega(t) - \left(b \cdot p_{BM} \cdot \frac{B_{ia}(t) + B_{ij}(t)}{B_T(t)} + \mu_M\right) M_s(t) \\
&M_e'(t) = b \cdot p_{VB} \cdot \frac{M_i}{B_T} \cdot M_s(t) - (\theta_M + \mu_M) M_e(t) \\
&M_i'(t) = \theta_M \cdot M_e(t) - \mu_M M_i(t) \\
&B_{sa}'(t) = -\left(\mu_B + b \cdot p_{MB} \cdot \frac{M_i(t)}{B_T(t)}\right) B_{sa}(t) \\
&B_{ea}'(t) = b \cdot p_{MB} \cdot \frac{M_i(t)}{B_T(t)} \cdot B_{sa}(t) - (\mu_B + \theta_B) B_{ea}(t) \\
&B_{ia}'(t) = \theta_B \cdot B_{ea}(t) - (\mu_B + \sigma_B) \cdot B_{ia}(t) \\
&B_{ra}(t) = \sigma_B \cdot B_{ia}(t) - \mu_B \cdot B_{ra}(t) \\
&B_{sj}'(t) = \gamma \cdot B_a - \left(\mu_{Bj} + b \cdot p_{MB} \cdot \frac{M_i(t)}{B_T(t)}\right) B_{sj}(t) \\
&B_{ej}'(t) = b \cdot p_{MB} \cdot \frac{M_i(t)}{B_T(t)} \cdot B_{sj}(t) - (\mu_{Bj} + \theta_B) B_{ej}(t) \\
&B_{ij}'(t) = \theta_B \cdot B_{ej}(t) - (\mu_{Bj} + \sigma_B) B_{ij}(t) \\
&B_{rj}(t) = \sigma_B \cdot B_{ij}(t) - \mu_{Bj} B_{rj}(t)
\end{aligned}
$$

Where B_T is the total avian population and B_a is the number of adult birds. All parameters are described in Table 1 in the main text.

Mosquito death rate (μ_M)

The mortality rate of adult female mosquitoes has been taken as the function of temperature suggested in [2], multiplied by the average increase factor found in [3].

Probability of WNV transmission from bird to vector (p_{BM})

In [4] the authors compute this rate for three different temperatures, namely 18, 23 and 28°C. We modeled this probability as a function of temperature following the approach presented in [3]. As shown in Figure S3, our proposed function fits well the observed values.

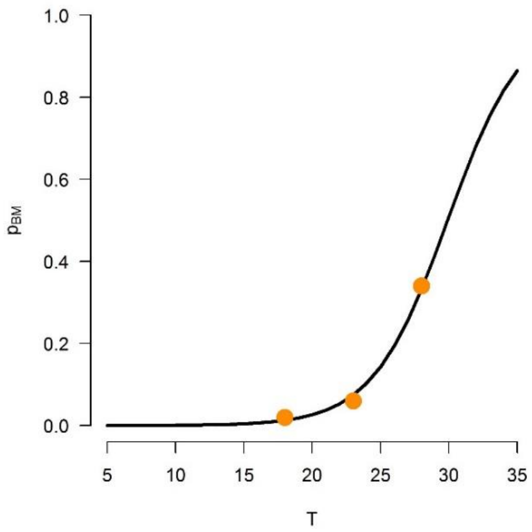

Supplementary Figure S3. Probability of WNV transmission from bird to vector (p_{BM}). Orange dots: observed laboratory data [4].

Additional results

Predicted seroprevalence

From Figure S4 it is clear that the two different initial assumptions (model B and M) produce very similar attack rates in the avian community. Unsurprisingly, with model B about 10% of the birds are immune to the virus during the earlier months of the mosquito breeding season.

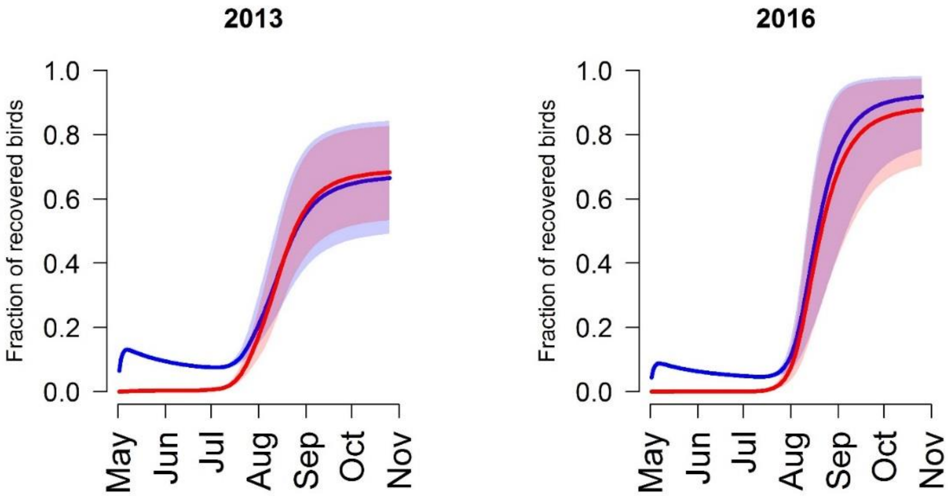

Supplementary Figure S4. Avian seroprevalence. Predicted fraction of birds that became immune after infection $(B_{ra}+B_{rj})/B_T$. Blue: model B; Red: model M. Solid lines: average values; shaded regions: 95% credible interval.

Human fit without model

For each week w, there are p_w positive pools out of n_w analyzed, with an average size (number of mosquitoes per pool) m_w. By dividing $p_w/n_w/m_w$ we can obtain an average WNV prevalence δ_W in the mosquito population for week w, together with 95%CI by performing a binomial test. Similarly to the procedure presented in the main text, we predicted the number of reported WNV human infections N_w from a Poisson($R \cdot \rho \cdot \delta_W \cdot M(w)$), where R is the ratio between the cluster area and trapped area *A*, *M*(w) is the average interpolated weekly mosquito abundance, δ_W is the mosquito prevalence drawn from the distributions computed as explained previously and ρ is a free rescaling parameter, estimated with a MCMC approach applied to the Poisson likelihood of observing the recorded infections, given the model predictions, by modelling simultaneously the two considered years. In this case, we can interpret ρ as a product of the mosquito biting rate on humans, the probability of virus transmission to humans per infectious bite, the probability of symptoms development and the reporting rate.

As shown in Figure S5, this simpler modelling approach fits very poorly the observed data. In particular, it overestimates the number of infections at the beginning of the season. Its associated DIC value is 93.7, which is much higher than the ones shown in Table 3.

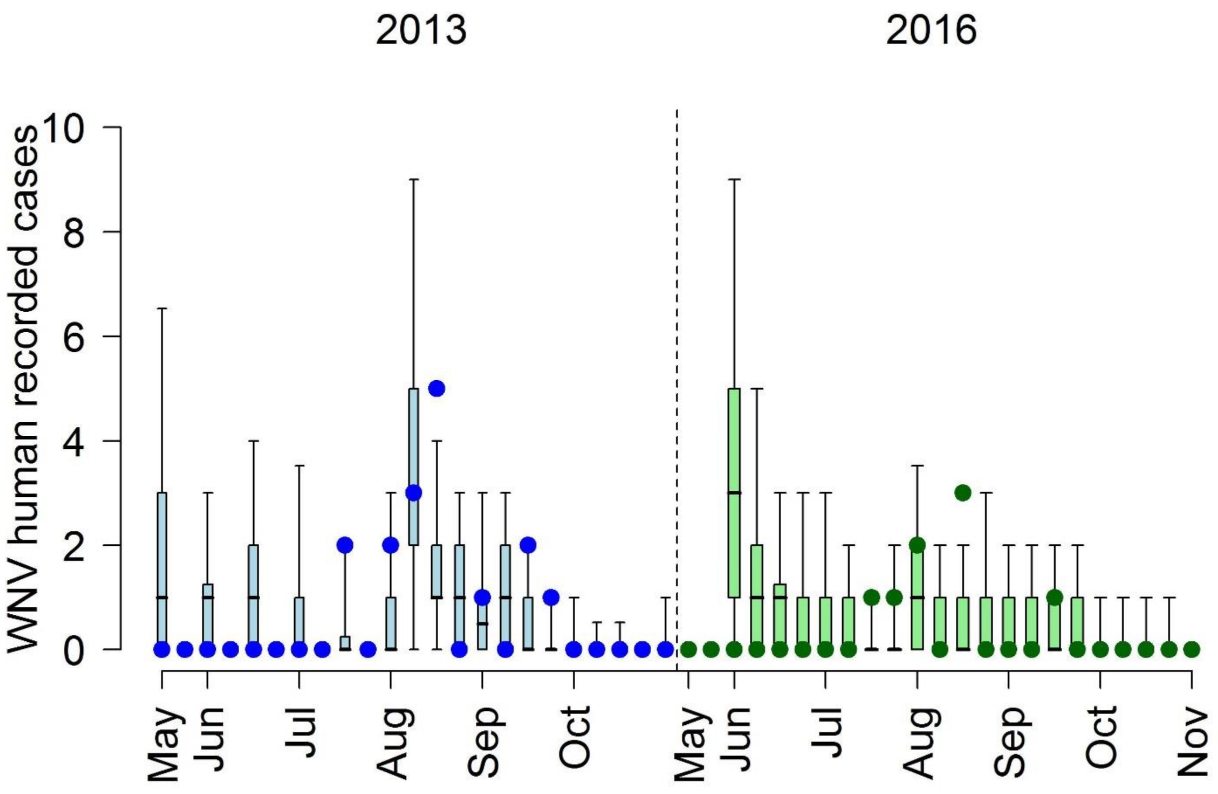

Supplementary Figure S5. Human infections. Notified human symptomatic cases in 2013 (blue) and 2016 (green). Filled dots: observed WNV symptomatic cases per week of symptoms onset. Boxplots (median, quartiles and 95% quantiles): cases predicted without the model presented in the main text.

Sensitivity analysis

We explored how different values for the model constant parameters might affect the model results. Precisely, for each parameter $p \in \Omega = \{p_{MB}, \sigma_B, \theta_B, \mu_B, \mu_{BJ}, \gamma)$ we run 100 MCMCs with p drawn from a normal distribution $N\left(\bar{p}, \left(\frac{\bar{p}}{10}\right)^2\right)$ where $\bar{p}$ is the parameter average value as reported in Table 1 in the main text. For instance, to assess if different p_{MB} values might affect our results, we run 100 samplings from N(0.94, 0.094^2) and, for each extracted parameter value, we fitted the observed data with models B and M by MCMC, as explained in the main text, and subsequently rerun the simulations to investigate whether such parameter perturbations affected substantially the predicted avian and vector prevalences.

As shown in Figures S6-S8, changes in the parameters in Ω do not substantially alter the estimated prevalence for both populations. In particular, we can note that the temporal dynamics is not varied whereas the magnitude might change slightly. Overall, such plots are comparable to the ones presented in the main text (Figure 3). For instance, the median of the 5% credible interval of the 2013 mosquito population is, at its peak, between 2.7 and $3.5 \cdot 10^{-3}$ when the bird susceptibility p_{MB} is changed (first line and column of Figure S7), similarly to what is reported in panel C in Figure 3 in the main text (lower boundary of the shaded area).

Table S1 reports the frequency (in percentage) of when model M performs better than model B, i.e. when the DIC value associated to model M is lower. This analysis corroborates our findings, as it is clear that usually model M outscores model B. We found that ΔDIC (the difference between the two DIC values) is slightly negatively correlated with two parameters in Ω, namely p_{MB} and γ, meaning that higher (lower) values of the avian susceptibility or fertility decrease (increase) the DIC difference between the two models. As there is no or low correlation between ΔDIC values and the perturbed parameters, we can conclude that model B might outscore model M probably due to stochastic reasons.

From Tables S2 and S3 we can note that the estimated distributions of the free model parameters are usually uncorrelated with the perturbed parameters, except for few cases when, although significant (p-value<0.05), the correlation coefficient is often lower than 0.3. The biting rate is more sensitive to changes in the parameters in Ω: for instance, higher avian fertility rates are associated to higher average estimated biting rates.

Overall, from Figures S9 and S10 it is clear that changes in the parameters in Ω do not produce substantial differences in the average estimated parameters with respect to the values presented in Table 2 in the main text. In addition, from Figure S10 we can note that model M usually fits worse than model B (empty dots) when the estimated average initial avian densities (B_0(2013), B_0(2016)) and biting rate (*b*) lie out of the cluster that includes the most frequent parameter estimations.

Finally, we can conclude that changes in the model constant parameters produce very small variations in model simulations. Moreover, such variations do not alter substantially the estimated values for the free parameters. Model M usually outscores model B and we can remark that, when assuming WNV is re-activated with infectious birds (blue lines of the last two columns in Figures S6-S8), the highest avian prevalence is always predicted to occur in spring, contrary to observation.

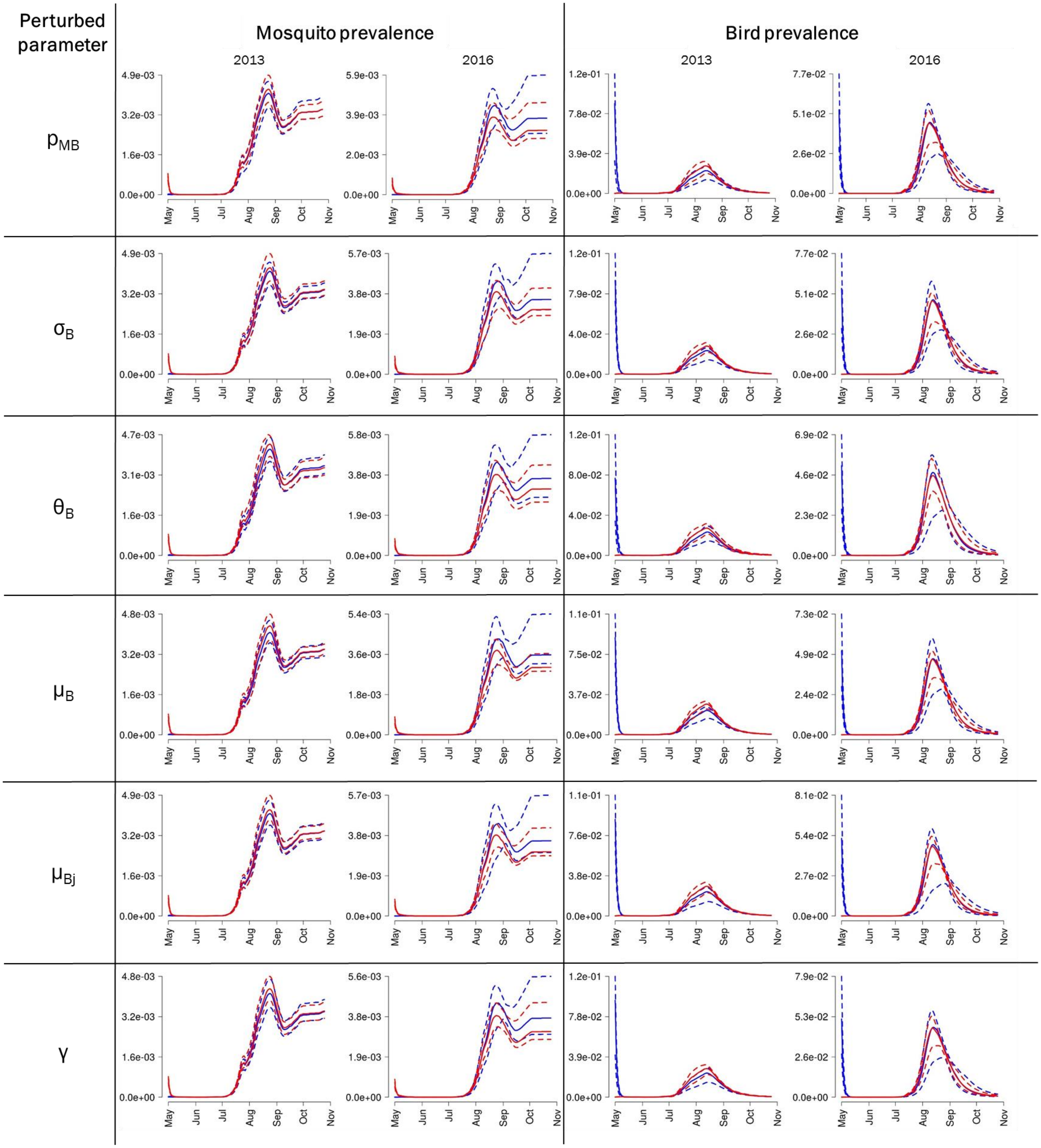

Supplementary Figure S6. Sensitivity analysis: median model output. Median (continuous lines) and 95% credible intervals of the median mosquito (first and second column) and avian (third and fourth column) prevalence as predicted by the model according to each perturbed parameter p_{MB}, δ_B, θ_B, μ_B, μ_{Bj}, γ (respectively from the first to the last row).

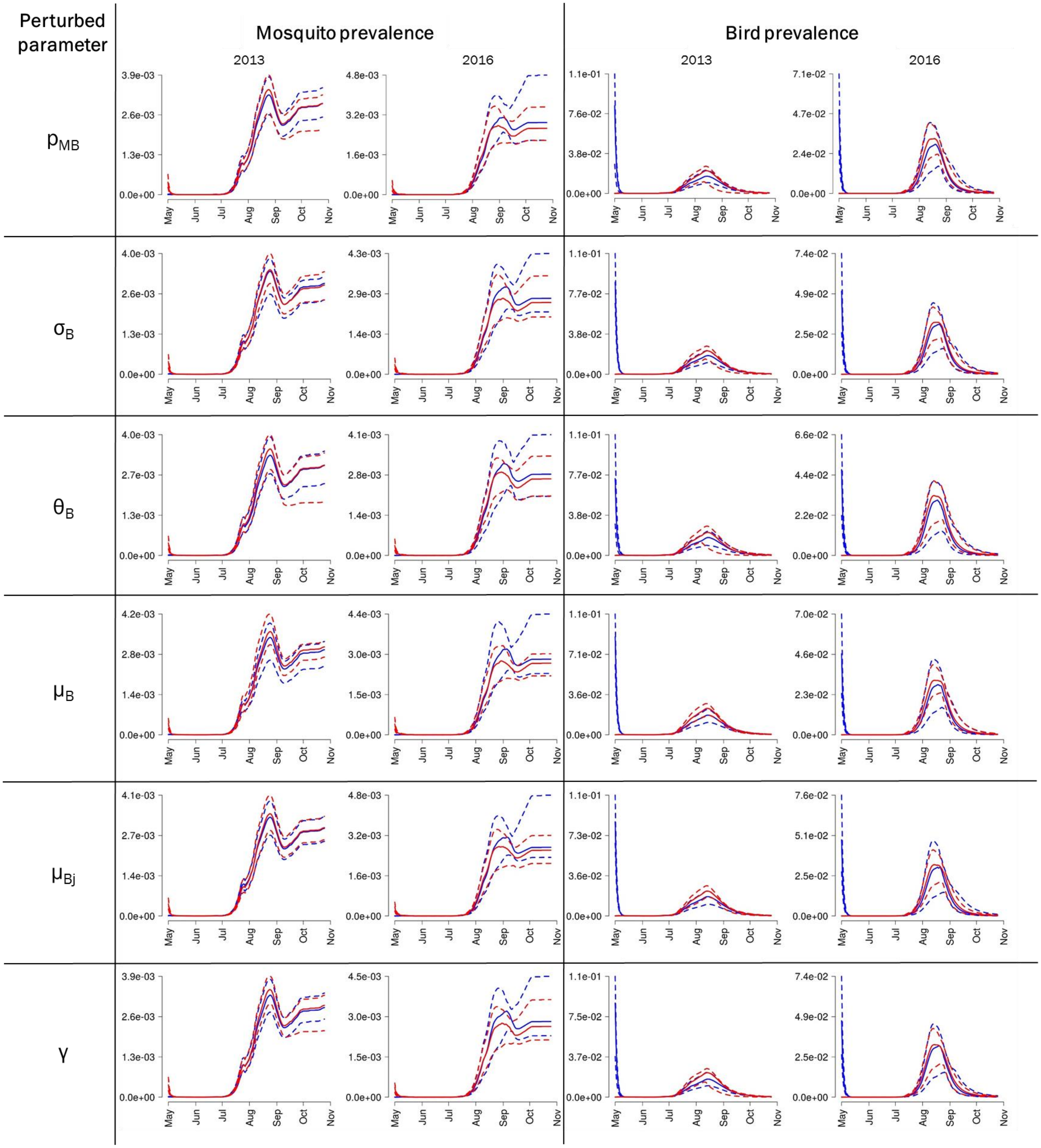

Supplementary Figure S7. Sensitivity analysis: 5%CI model output. Median (continuous lines) and 95% credible intervals of the 5% credible interval of the mosquito (first and second column) and avian (third and fourth column) prevalence as predicted by the model according to each perturbed parameter p_{MB}, δ_B, θ_B, μ_B, μ_{Bj}, γ (respectively from the first to the last row).

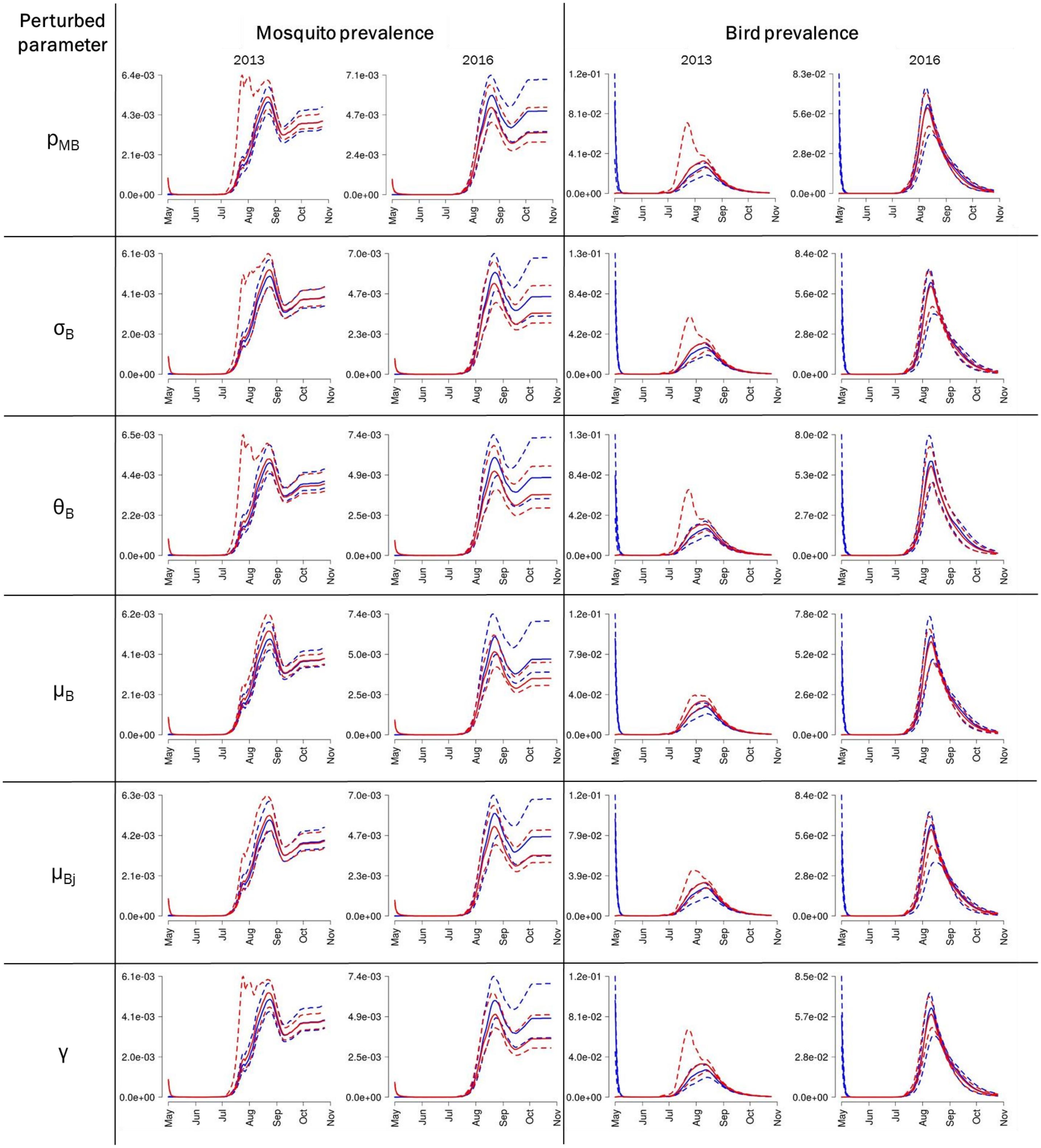

Supplementary Figure S8. Sensitivity analysis: 95%CI model output. Median (continuous lines) and 95% credible intervals of the 95% credible interval of the mosquito (first and second column) and avian (third and fourth column) prevalence as predicted by the model according to each perturbed parameter p_{MB}, δ_B, θ_B, μ_B, μ_{Bj}, γ (respectively from the first to the last row).

Supplementary Table S1. First column: the frequency of when Model M fits the observed data better than model B for each perturbed parameter in Ω. Last column: Pearson correlation coefficient χ between ΔDIC and the perturbed parameter (reported only if statistically significant). ΔDIC=DIC(Model B)-DIC(Model M).

**Perturbed parameter**	**ΔDIC>0**	χ
p_{MB}	92%	-0.21
δ_B	84%	-
θ_B	85%	-
μ_B	89%	-
μ_{Bj}	82%	-
γ	79%	-0.27

Supplementary Table S2. Coefficient of the Pearson correlation between the average of the estimated free model B parameters and the perturbed parameters in Ω. Only statistically significant (p-value<0.05) values are reported.

**Perturbed parameter**	***p*(2013)**	***p*(2016)**	**B_0(2013)**	**B_0(2016)**	***b***
p_{MB}	0.63	-0.27	-	-0.22	-0.38
δ_B	-	-	-	-	-
θ_B	-	0.41	-0.43	-0.41	-
μ_B	0.25	-	-	-	-
μ_{Bj}	-	-	-	-	-
γ	-	-	-	-	0.25

Supplementary Table S3. Coefficient of the Pearson correlation between the average of the estimated free model M parameters and the perturbed parameters in Ω. Only statistically significant (p-value<0.05) values are reported.

**Perturbed parameter**	***p*(2013)**	***p*(2016)**	**B_0(2013)**	**B_0(2016)**	***b***
p_{MB}	-0.22	-	-	-	-0.23
δ_B	-	-	-	-	-
θ_B	-0.23	-	-	-	0.32
μ_B	-	-	-	-	-
μ_{Bj}	-	0.39	-	-	-0.25
γ	-	-	0.25	0.24	0.45

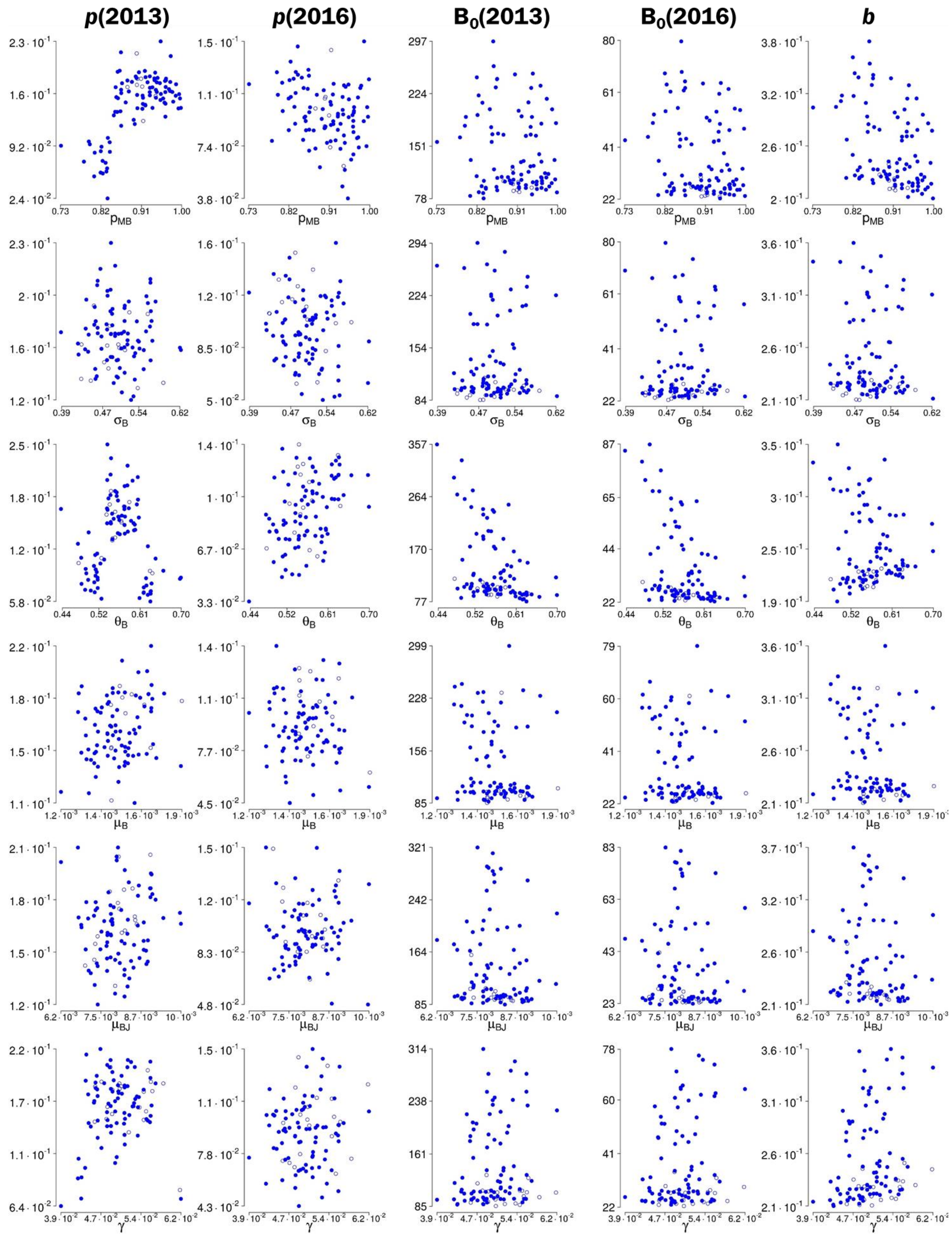

Supplementary Figure S9. Sensitivity analysis for model B. Average estimated values for the free model parameters *p*(2013), *p*(2016), B_0(2013), B_0(2016), *b* (respectively from the first to the last column) according to each perturbed parameter p_{MB}, δ_B, θ_B, μ_B, μ_{Bj}, γ (respectively from the first to the last row). Empty dots indicate when the DIC difference between model B and model M is negative (i.e. model B fits better the observed entomological data).

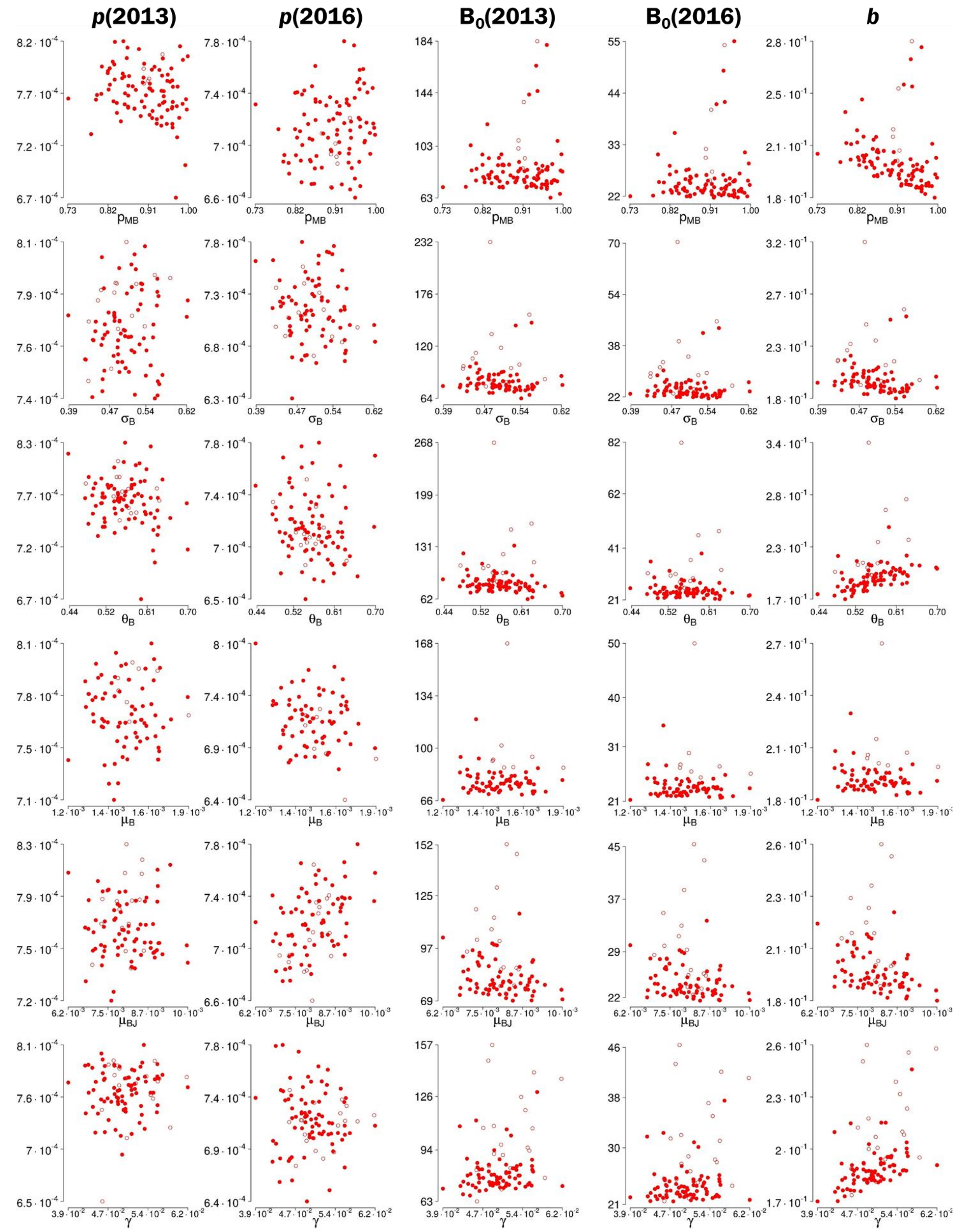

Supplementary Figure S10. Sensitivity analysis for model M. Average estimated values for the free model parameters *p*(2013), *p*(2016), B_0(2013), B_0(2016), *b* (respectively from the first to the last column) according to each perturbed parameter p_{MB}, δ_B, θ_B, μ_B, μ_{Bj}, γ (respectively from the first to the last row). Empty dots indicate when the DIC difference between model B and model M is negative (i.e. model B fits better the observed entomological data).

References

1. Arpa Veneto Dati Ambientali. Available: http://www.arpa.veneto.it/dati-ambientali (2018)
2. Ciota, A. T., Matacchiero, A. C., Kilpatrick, A. M. & Kramer, L. D. The Effect of Temperature on Life History Traits of Culex Mosquitoes. *J. Med. Entomol.* **51,** 55–62 (2014).
3. Marini, G. *et al.* The Role of Climatic and Density Dependent Factors in Shaping Mosquito Population Dynamics: The Case of Culex pipiens in Northwestern Italy. *PLOS ONE* **11,** e0154018 (2016).
4. Vogels, C. B. F., Fros, J. J., Göertz, G. P., Pijlman, G. P. & Koenraadt, C. J. M. Vector competence of northern European Culex pipiens biotypes and hybrids for West Nile virus is differentially affected by temperature. *Parasites & Vectors* **9,** 393 (2016).
